# No delayed intracranial hemorrhage in head injury patients on oral anticoagulants and with normal CT: a retrospective study of 215 patients

**DOI:** 10.1186/s13049-026-01617-1

**Published:** 2026-04-29

**Authors:** Martin Aase Bahr, Håvard Visnes, Tor Brommeland

**Affiliations:** 1 Department of Orthopedic Surgery, Sørlandet Hospital, Kristiansand, Norway; 2 Department of Neurosurgery, Oslo University Hospital, Oslo, Norway

## Abstract

**Background:**

The current Scandinavian Neurotrauma Committee’s (SNC) guidelines mandate 24-hour hospital observation for head injury patients on anticoagulation therapy even with a normal head CT to detect delayed hemorrhages. However, emerging evidence suggests the incidence of delayed intracranial hematoma in this context is very low. A new protocol was developed at Sørlandet Hospital Kristiansand allowing for immediate discharge of patients on anticoagulation therapy with Glasgow Coma Scale (GCS) 14–15 and normal head CT. This study evaluates the management, incidence of delayed intracranial hemorrhage and re-contact rates in anticoagulated patients with a head injury and normal CT.

**Methods:**

A retrospective review of all patients who sustained a head injury and underwent a CT scanning at Sørlandet Hospital Kristiansand, Norway from April 1, 2024, to December 12, 2025, was performed. Patients were included in the study if they were taking oral anticoagulation of any type and had a normal CT scanning of the head. Demographic data, mechanism of injury, GCS on admission, discharge or admission decisions, re-contact with the hospital, neurosurgical intervention and mortality were registered. Delayed intracranial hemorrhage was defined as any intracranial hematoma not evident on the initial CT but detected on a subsequent CT scan. Clinically relevant delayed hemorrhage was considered as a hematoma requiring re-admission, neurosurgical intervention, or causing death.

**Results:**

A total of 795 patients underwent head CT with a median age of 74 years (3–99 years). Of these, 232 patients were taking oral anticoagulation of which 215 had normal CT scans and composed our study group. Median age in the study group was 83 years (33–97 years). GCS on admission was 14–15 in 223/232 (96.1%) of patients. None developed delayed intracranial hemorrhage during observation or follow-up. No patients were transferred for neurosurgical intervention or experienced any clinically significant deterioration due to delayed hemorrhage.

**Conclusion:**

In this cohort of anticoagulated head injury patients with normal head CT, the incidence of delayed intracranial hemorrhage was zero. Our findings suggest that routine 24-hour observation of all such patients may offer limited benefits, and that a more selective management approach could be justified.

## Introduction

The widespread use of oral anticoagulants in the past decade now exposes an increasing number of patients sustaining head trauma to the risk of an intracranial hematoma [1, 2]. Several guidelines have been published to aid clinicians handle patients with mild, moderate, or severe head injuries in daily practice [3–6]. The Scandinavian Neurotrauma Committee (SNC) published guidelines for the initial management of patients with head injury in 2013 [3]. These guidelines call for a mandatory head CT of all patients on oral anticoagulation therapy sustaining a head injury. In addition, an in-hospital observation of minimum 24 h is recommended to avoid missing delayed intracranial hematomas, even if the initial head CT is normal.

Emerging evidence suggests that delayed intracranial hematomas (ICH) after normal initial CT scans are rare, ranging between 0.08 and 2.3% [7, 8]. Delayed intracranial hematoma is defined as an ICH within 30 days of head trauma not identified on the first CT scan performed within 24 h of the trauma and without known repeat head trauma occurring since the preceding scan [8].

These findings challenge the benefit of routine admission and observation for all head trauma patients on anticoagulation therapy with a normal CT. Despite growing evidence that delayed ICH is rare, Scandinavian guidelines still mandate admission. The orthopedic department of Sørlandet Hospital Kristiansand, Norway oversees initial clinical and radiological examinations of all patients with a head trauma from a catchment area of approximately 155.000 inhabitants. Patients with head injuries requiring neurosurgical observation or intervention are transferred to Oslo University Hospital. The SNC guidelines have been routinely applied by the on-call orthopedic surgeons since 2013, and the department evaluates head trauma patients daily.

Considering the emerging evidence our department implemented a protocol change in 2024. Patients on oral anticoagulation with a normal CT scan and a Glasgow Coma Scale of 14–15, without neurological deficits, could now be discharged directly from the emergency department.

The aim of this study is to evaluate the clinical and radiological outcomes following this change in practice, with particular emphasis on patient safety.

## Methods

We conducted this retrospective observational study at Sørlandet Hospital Kristiansand, a regional hospital serving a population of 155,000. The study period spanned from April 2024 to September 2025. The study was performed as a quality-of-care evaluation following a local modification of the institutional management protocol for head-injured patients receiving oral anticoagulation therapy.

Prior to March 2024, patient management followed the Scandinavian Neurotrauma Committee (SNC) guidelines recommending hospital admission and 24-hour observation for patients on oral anticoagulation after head trauma despite a normal initial CT scan. In March 2024, the local guideline was modified such that patients receiving oral anticoagulation therapy with a mild head injury and a normal initial head CT could be discharged home from the emergency department without mandatory in-hospital observation, provided that no other clinical indications for admission were present. An overview of the modified protocol is provided in Table 1.

A written, modified version of the SNC head injury guidelines was created allowing the attending orthopedic surgeon to discharge anticoagulated patients if the CT scan was reported as normal by a radiologist and the clinical evaluation satisfactory. If admitted, patients were allowed to leave the hospital after < 24 h if their general condition allowed this. Upon discharge, patients and/or the caregiver were informed orally and in writing about the risk of delayed intracranial hemorrhage and the need for renewed contact with the hospital in case of clinical deterioration. If the initial CT scan was normal anticoagulation was continued and radiological or clinical controls not arranged.

**Fig. 1:**
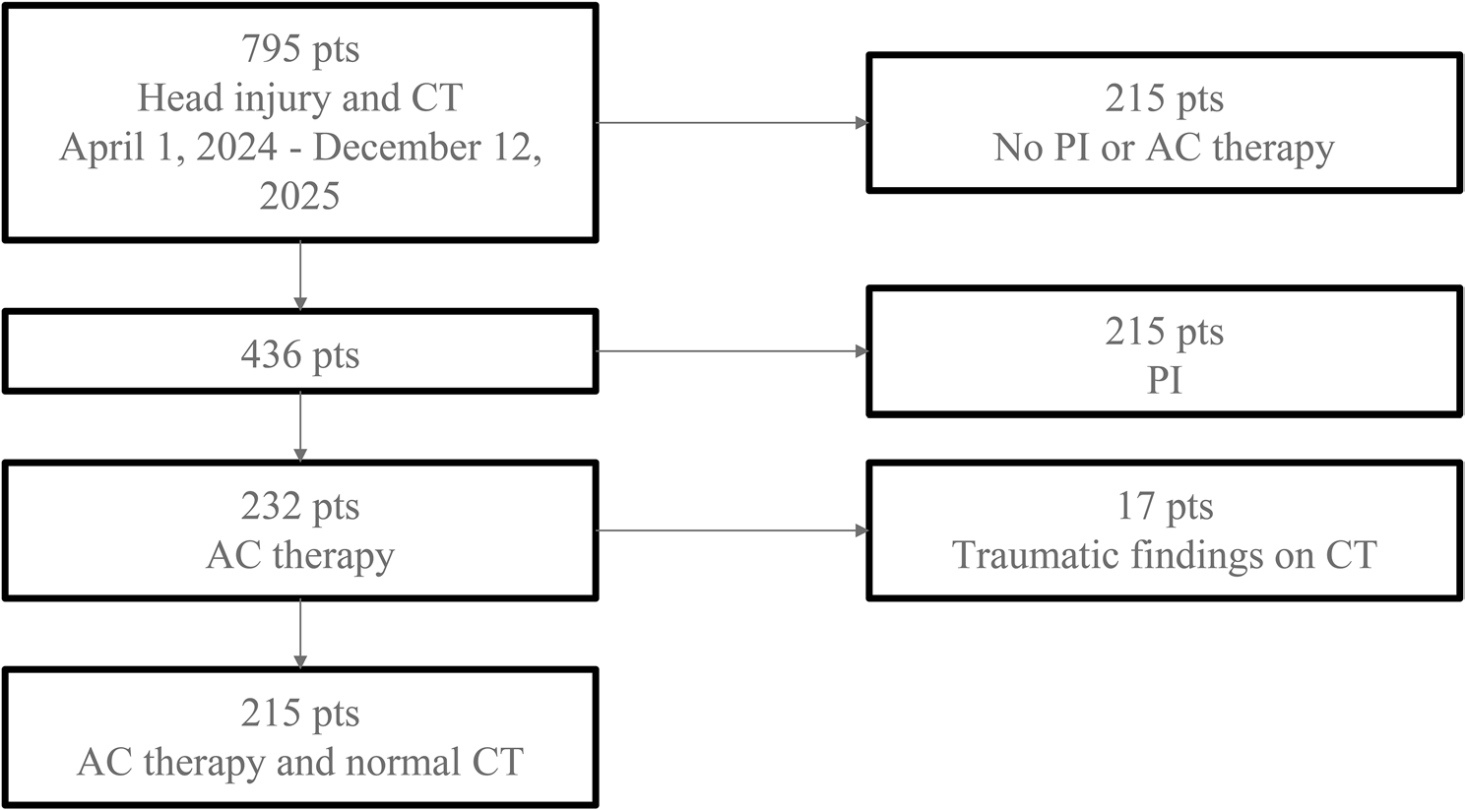
Flowchart depicting patient inclusion. AC: Anticoagulation, PI: Platelet inhibitor

We included all adult patients (≥ 18 years) presenting to the emergency department during the study period with head trauma while receiving oral anticoagulation therapy. Eligible patients were identified through electronic hospital records and radiology databases. Patients receiving dual antithrombotic therapy (anticoagulant plus antiplatelet), patients on antiplatelet therapy alone, and patients without confirmed oral anticoagulation use at the time of injury were excluded. See Fig. 1 for flowchart depicting patient inclusion.

Patients receiving dual antithrombotic therapy were excluded because combined anticoagulant and antiplatelet treatment represents a clinically distinct population with substantially higher hemorrhagic risk [9, 10].

Clinical data were extracted from electronic medical records and the hospital radiology information system. The following variables were collected: Age and sex, mechanism of injury, Glasgow Coma Scale (GCS) score on admission, findings on initial head CT, hospital admission, repeat imaging, emergency department revisits, neurosurgical intervention, mortality within 30 days.

The primary objective was to evaluate the safety of early discharge by determining the incidence of delayed intracranial hemorrhage in anticoagulated head injury patients with a normal CT.

The secondary objectives and outcomes were to assess clinically relevant outcomes including neurosurgical intervention and mortality within 30 days.

The primary outcome was delayed intracranial hemorrhage, defined as any intracranial bleeding not present on the initial head CT but detected on a subsequent CT scan or clinical presentation within 30 days after injury.

Follow-up data were obtained through review of hospital electronic records, including emergency department revisits, repeat imaging, hospital admissions, and mortality within 30 days of the index trauma. As Sørlandet Hospital Kristiansand is the sole emergency hospital for the region, clinically significant delayed intracranial hemorrhages requiring medical attention would be expected to present at this institution.

There were no missing data during the journal review. Variables relevant to the primary outcome were available for all included patients. There were no known missing values in secondary descriptive values.

Descriptive statistics were used to summarize patient characteristics and outcomes. Continuous variables are presented as medians with ranges, and categorical variables as counts and percentages. Incidence estimates are reported with 95% confidence intervals (CI).

The study was conducted as a quality-of-care evaluation and approved by the institutional data protection officer. Formal ethical approval was waived according to national regulations.

An overview of the modified protocol is provided in Table 1.

**Table 1:** Discharge criteria

GCS 14 or 15 No neurological deficit No intoxication No severe symptoms (headache, vomiting) No dual therapy (anticoagulation and antiplatelet treatment)
No VP-shunt No observed seizures CT normal and final report by radiologist in writing Patient or caretaker approval Oral and written information for renewed hospital contact

GCS: Glasgow Coma Scale VP: Ventriculoperitoneal

## Results

A total of 795 patients were clinically evaluated and examined with a head CT during the study period. Oral anticoagulation therapy was taken by 232 patients of which 215 had normal CT scans and were included in the study. 17 patients (7.9%) had traumatic intracranial findings on the initial CT scan. Results are summarized in Table 2. The median age of the study population was 83 years (33–97 years), and 43.7% were female. Most patients (90.7%) were older than 65 years. Fall from standing height was the mechanism of head injury in 203/215 (94.4%). GCS on admission was 14 or 15 in 207/215 (96.3%) patients. Time from injury to CT scanning was < 12 h in 195 (90.7%), 12–24 h in 8 (3.7%) and > 24 h in 12 (5.6%) of patients.

An immediate discharge to home or care-providing institution was allowed for 146 (67.9%) patients after clinical evaluation and confirmed normal CT scanning. Admission to the orthopedic department due to injury related complaints was provided for 27/215 (12.6%) patients while the remaining 22 (10.2%) were accepted in the medical wards for other, non-injury related conditions. None of the admitted patients were observed specifically for their head injuries or required to fast. Two patients had persistent headaches or dizziness after leaving the hospital and returned for repeated CT scans which demonstrated no intracranial hemorrhage. Thus, the observed rate of delayed intracranial hemorrhage out of 215 patients was 0% (95% CI 0–1.7%) No patients with an initially normal CT scan underwent later neurosurgical transfer or required intensive care due to head injury–related causes. Two patients died in hospital from hip fractures and heart failure, neither showing concomitant intracranial injury.

**Table 2:** Patient characteristics

	No (%)
Total patients	215
Median age (range, yrs)	83 (33–97)
Male: Female	121:94
GCS	
10–13	8 (3.7)
14–15	207 (96.3)
Type of AC therapy	
DOAC	198 (92.1)
Vitamin K antagonists	17 (7.9)
Management	
Discharge	146 (67.9)
Admission to orthopedic department	27 (12.6)
Admission to communal short stay unit observation	20 (9.3)
Admission to medical department	22 (10.2)
Time from injury to CT	
< 12 h	195 (90.7)
12–24 h	8 (3,7)
> 24 h	12 (5.6)

GCS: Glasgow Coma Scale DOAC: Direct Oral Anticoagulants

## Discussion

This is the first study in Scandinavia to report outcomes under a non-observation protocol. Of 215 patients on oral anticoagulation sustaining a head injury, 67.9% were discharged immediately after satisfactory clinical evaluation and confirmed normal CT scanning. There were no cases of clinically relevant delayed intracranial hemorrhage. Admission was provided for the remaining patients due to assorted reasons such as orthopedic fractures or medical conditions requiring hospitalization. Two patients were re-examined clinically and radiologically due to persistent symptoms related to their head injury of which none demonstrated delayed intracranial hemorrhage on repeat CT scanning. The re-evaluation was day four and day five after primary examination. None of the patients were later re-hospitalized or transferred to a neurosurgical department for their initial head injury. No head-injury related deaths were registered.

It should be stressed that a clinical evaluation regarding the safety of immediate discharge was carefully assessed by the on-call orthopedic surgeon. All discharged patients were awake with GCS 14 or 15 with no neurological deficits. Patients were admitted if they declined to be discharged to their home, were intoxicated or had symptoms or clinical findings that warranted further investigations. For some patients discharge late at night was difficult and admission was approved for logistical reasons.

The head CT was investigated by the on-call radiologist and discharge was only approved when the final report was in writing. We consider this crucial to avoid missing delayed ICH. Small ICH as contusions in the frontobasal regions of the brain may be difficult to discover by physicians not accustomed to detailed investigations of head CT. Brain contusions may enlarge within hours of the initial trauma especially in patients on oral anticoagulation [11].

Our study indicates that the risk of delayed intracranial hemorrhage in patients on anticoagulation therapy is very low when the head CT is normal. These findings are in concordance with others. André et al. reported only 0.08% clinically relevant delayed bleeds in a Swedish cohort study of 2362 cases, 56% of those on DOAC [8]. Similarly, Puzio et al. found a 2.3% incidence of delayed hemorrhage in a systematic review and noted that associated mortality (~ 0.3%) and neurosurgical intervention rates (~ 0.1%) were extremely low [7]. Capsoni et al. investigated the overall occurrence of ICH in 1596 patients on oral anticoagulation and performed repeated mandatory CT scanning within 24 h of a negative CT [11]. The incidence of new ICH was 1.8% for DOAC and 2.6% for vitamin K-antagonists. None of these patients required neurosurgical interventions. Others have also reported that vitamin K-antagonists seem to have a higher risk of ICH than DOAC even on an initial CT scan after head trauma (Savioli et al. [12]). In our study, 92.1% were taking DOAC and the number of patients taking vitamin K antagonists was too small to perform any comparison. A recent Swedish study by Bergenfeldt et al. (2023) concluded that delayed hemorrhage after head trauma in anticoagulated patients is very rare and seldom necessitates any intervention [13]. Altogether, these observations challenge the benefit of routine prolonged observation for all anticoagulated head injury patients.

Despite this evidence, some guidelines like the SNC still recommend admitting all anticoagulated head trauma patients with a normal CT scan for a 24-hour observation [3]. More recent international guidelines reflect a changing approach: the UK’s National Institute for Health and Care Excellence (NICE) and French head injury guidelines no longer advocate mandatory observation after a negative initial CT in anticoagulated patients [14, 15]. Our findings support a re-evaluation of the obligatory observation policy and that a more selective approach may be justified.

The potential downsides of unnecessary hospital admissions are several. Economically, the average cost in Norway of 24-hours of inpatient care is 2216 euros [16]. The increased burden on public hospitals with an aging population is well documented and avoiding unnecessary admissions is important [17, 18]. Even short-term hospitalizations may harm elderly, frail patients including the risk of inpatient falls and delirium [19, 20]. In line with this, Benedetti et al. argued that a 24-hour observation period for mild TBI patients on anticoagulation should not be routinely recommended without clear evidence of benefit [21].

There are several limitations to this study. First, the retrospective design introduces a risk of selection bias. The sample size is modest, raising statistical power concerns; if the true incidence of delayed ICH is on the order of ~ 0,08%, such a rare event could go undetected in a cohort of this size [8]. Although no delayed hemorrhages were observed in our population, the confidence interval indicates that the true incidence could be as high as 1,8%. It is debatable what level of residual risk is clinically acceptable. There are also potential diagnostic uncertainties, studies have shown a false-negative rate of ~ 2% for initial head CT interpretations [22]. The reliability of Glasgow Coma Scale assessment in our cohort of very elderly patients is another concern. Cognitive impairment, or communication difficulties can make GCS scoring less accurate in older patients [23].

Further studies with larger patient samples are needed to verify our findings and refine criteria for which anticoagulated head injury patients truly benefit from hospital admission and prolonged observation.

## Conclusion

In this single-center series of 215 anticoagulated head injury patients, we observed no clinically relevant delayed intracranial hemorrhages after an initially normal CT scan. These results suggest that mandatory 24-hour in-hospital observation of all such patients offers limited benefit. A more selective management approach based on individual risk assessment would better balance patient safety and resource utilization.

## Data Availability

The processed data is connected to the patient ID, stored on hospital servers, and can therefore not be shared openly. The data that support these findings are available from the corresponding author upon reasonable request. The data are located in controlled data storage at Sørlandet hospital.

## References

[1] Navar AM, Kolkailah AA, Overton R, Shah NP, Rousseau JF, Flaker GC, et al. Trends in Oral Anticoagulant Use Among 436 864 Patients With Atrial Fibrillation in Community Practice, 2011 to 2020. J Am Heart Assoc. 2022;11(22):e026723. 36346063 10.1161/JAHA.122.026723 PMC9750070

[2] Iyer GS, Tesfaye H, Khan NF, Zakoul H, Bykov K. Trends in the Use of Oral Anticoagulants for Adults With Venous Thromboembolism in the US, 2010–2020. JAMA Netw Open. 2023;6(3):e234059–e. 36947039 10.1001/jamanetworkopen.2023.4059 PMC10034573

[3] Unden J, Ingebrigtsen T, Romner B, Scandinavian Neurotrauma C. Scandinavian guidelines for initial management of minimal, mild and moderate head injuries in adults: an evidence and consensus-based update. BMC Med. 2013;11:50. 23432764 10.1186/1741-7015-11-50 PMC3621842

[4] Excellence NIfHaC. Head injury: assessment and early management. London: National Institute for Health and Care Excellence (NICE); 2023. p. NG232. Report No. 37289922

[5] Prevention CfDCa. Mild traumatic brain injury management guideline. Atlanta, GA: Centers for Disease Control and Prevention (CDC); 2023.

[6] Trauma EAftSo. Evaluation and management of mild traumatic brain injury: an Eastern Association for the Surgery of Trauma practice management guideline. J Trauma Acute Care Surg. 2012;73(5):S307–14. 23114486 10.1097/TA.0b013e3182701885

[7] Puzio TJ, Murphy PB, Kregel HR, Ellis RC, Holder T, Wandling MW. Delayed intracranial hemorrhage following blunt head trauma while on direct oral anticoagulants: a systematic review and meta-analysis. J Am Coll Surg. 2021;232:1007–16. 33766725 10.1016/j.jamcollsurg.2021.02.016 PMC8722268

[8] Andre L, Bjorkelund A, Ekelund U, Vedin T, Bjork J, Forberg JL. The prevalence of clinically relevant delayed intracranial hemorrhage in head trauma patients treated with oral anticoagulants is very low: a retrospective cohort register study. Scand J Trauma Resusc Emerg Med. 2024;32(1):42. 38730480 10.1186/s13049-024-01214-0 PMC11084042

[9] Farrokh S, Nalleballe K, Onteddu S, Suarez JI, Bösel J, Shah VA. Bleeding Risk With Combining Antiplatelets and Anticoagulants for Secondary Stroke Prevention: A Propensity Score–Matched Analysis. J Am Heart Association. 2025;14(16):e042767. 10.1161/JAHA.125.042767 PMC12748079 40831308

[10] Hansen ML, Sørensen R, Clausen MT, Fog-Petersen ML, Raunsø J, Gadsbøll N, et al. Risk of Bleeding With Single, Dual, or Triple Therapy With Warfarin, Aspirin, and Clopidogrel in Patients With Atrial Fibrillation. Arch Intern Med. 2010;170(16):1433–41. 20837828 10.1001/archinternmed.2010.271

[11] Capsoni N, Carpani G, Tarantino F, Gheda S, Cugnod JM, Lanfranchi S, et al. Incidence and risk factors for delayed intracranial hemorrhage after mild brain injury in anticoagulated patients: a multicenter retrospective study. Scand J Trauma Resusc Emerg Med. 2025;33(1):26. 39930444 10.1186/s13049-025-01337-y PMC11808940

[12] Savioli G, Ceresa IF, Luzzi S, Gragnaniello C, Giotta Lucifero A, Del Maestro M, et al. Rates of intracranial hemorrhage in mild head trauma patients presenting to emergency department and their management: a comparison of direct oral anticoagulant drugs with vitamin K antagonists. Med (Kaunas). 2020;56(6). 10.3390/medicina56060308 PMC7353902 32585829

[13] Bergenfeldt H, Forberg JL, Lehtinen R, Anefjall E, Vedin T. Delayed intracranial hemorrhage after head trauma seems rare and rarely needs intervention-even in antiplatelet or anticoagulation therapy. Int J Emerg Med. 2023;16(1):54. 37667208 10.1186/s12245-023-00530-z PMC10476369

[14] National Institute for Health and Care Excellence (NICE). Head injury: assessment and early management. 2025.

[15] Gil-Jardine C, Payen JF, Bernard R, Bobbia X, Bouzat P, Catoire P, et al. Management of patients suffering from mild traumatic brain injury 2023. Anaesth Crit Care Pain Med. 2023;42(4):101260. 37285919 10.1016/j.accpm.2023.101260

[16] Helsedirektoratet. Kostnader, produktivitet og økonomisk status i spesialisthelsetjenesten. Rapport IS-3011. Oslo: Helsedirektoratet; 2021.

[17] Van den Heede K, Bouckaert N, Van de Voorde C. The impact of an ageing population on the required hospital capacity: results from forecast analysis on administrative data. Eur Geriatr Med. 2019;10(5):697–705. 34652701 10.1007/s41999-019-00219-8

[18] Keeble E, Roberts HC, Williams CD, Van Oppen J, Conroy SP. Outcomes of hospital admissions among frail older people: a 2-year cohort study. Br J Gen Pract. 2019;69(685):e555–60. 31308000 10.3399/bjgp19X704621 PMC6650131

[19] Sousa P, Uva AS, Serranheira F, Uva MS, Nunes C. Patient and hospital characteristics that influence incidence of adverse events in acute public hospitals in Portugal: a retrospective cohort study. Int J Qual Health Care. 2018;30(2):132–7. 29309608 10.1093/intqhc/mzx190 PMC5890867

[20] San Jose-Saras D, Vicente-Guijarro J, Sousa P, Moreno-Nunez P, Aranaz-Andres JM, Health Outcomes Research Group of the Instituto Ramon y Cajal de Investigacion S. Inappropriate hospital admission as a risk factor for the subsequent development of adverse events: a cross-sectional study. BMC Med. 2023;21(1):312. 37592294 10.1186/s12916-023-03024-0 PMC10433586

[21] Benedetti S, Benedetti MD, Tomasi D, Palmisano G, Calcagno S, Bianchi S, et al. In old anticoagulated patients with mild traumatic brain injury, a 24-h observation period should not be recommended without evidence of a clear benefit: a retrospective study of delayed hemorrhagic versus iatrogenic complications. Intern Emerg Med. 2024;19(2):523–34. 37812308 10.1007/s11739-023-03435-0

[22] Erly WK, Ashdown BC, Richard W, Lucio I, Carmody RF, Seeger JF, Alcala JN. Evaluation of Emergency CT Scans of the Head: Is There a Community Standard? Am J Roentgenol. 2003;180(6):1727–30. 12760951 10.2214/ajr.180.6.1801727

[23] Salottolo K, Levy AS, Slone DS, Mains CW, Bar-Or D. The effect of age on Glasgow Coma Scale score in patients with traumatic brain injury. JAMA Surg. 2014;149(7):727–34. 24899145 10.1001/jamasurg.2014.13

